# Age-Related Differences in Percentages of Regulatory and Effector T Lymphocytes and Their Subsets in Healthy Individuals and Characteristic STAT1/STAT5 Signalling Response in Helper T Lymphocytes

**DOI:** 10.1155/2015/352934

**Published:** 2015-10-07

**Authors:** Marija Holcar, Aleš Goropevšek, Alojz Ihan, Tadej Avčin

**Affiliations:** ^1^Department of Allergology, Rheumatology and Clinical Immunology, University Children's Hospital, University Medical Centre Ljubljana, Bohoričeva 20, SI-1525 Ljubljana, Slovenia; ^2^Department of Laboratory Diagnostics, University Medical Centre Maribor, Ljubljanska Ulica 5, SI-2000 Maribor, Slovenia; ^3^Faculty of Medicine, University of Maribor, Taborska Ulica 8, SI-2000 Maribor, Slovenia; ^4^Institute of Microbiology and Immunology, Faculty of Medicine, University of Ljubljana, Zaloška 4, SI-1000 Ljubljana, Slovenia; ^5^Department of Pediatrics, Faculty of Medicine, University of Ljubljana, Vrazov trg 2, SI-1000 Ljubljana, Slovenia

## Abstract

The dynamic process of the development of the immune system can in itself result in age-related immune malfunctions. In this study, we analysed lymphocyte subsets in the peripheral blood of 60 healthy donors, divided into groups of children, adolescents, and adults, focusing on effector (Teff) and regulatory (Treg) T lymphocytes and STAT1/STAT5 signalling response in helper T lymphocytes (Th) in adults, using flow cytometry. Our results demonstrate a decrease in the percentage of total Tregs and an increase in the percentage of total Teffs with age and a consequential immense increase in the Teff/Treg ratio. The increase of Teffs was most apparent in Th1, Th1Th17, and Th17CD161− subsets. Significant Th lymphocyte STAT1 expression differences were observed between children and adolescents, which were associated with the decrease in activated Tregs. Higher expression of STAT1 was found in FoxP3hi than in FoxP3low Th lymphocytes, while significant IL-2 induced STAT5 phosphorylation differences were found among the subsets of Th lymphocytes in adults. Our study demonstrates age-related changes in circulating Teff and Treg, as well as significant differences in STAT5/STAT1 signalling among FoxP3+ Th lymphocytes, providing new advances in the understanding of immunosenescence.

## 1. Introduction

The development, differentiation, and evolution of the immune system are dynamic processes evolving throughout the life of an individual. The immune system of a child begins to resemble that of an adult only after puberty under the influence of sex hormones, which promote full maturation of the immune system [1]. Alterations of the immune system continue with the gradual deterioration in the elderly, resulting in age-related immune dysfunctions (immunosenescence) such as increased malignancies, autoimmune disorders, susceptibility to both viral and bacterial infections, and diminished response to vaccination [2–5]. Analysis of lymphocyte subsets in peripheral blood is useful in the evaluation of the immune status of the investigated subject and can help to indicate the dynamics and cooperation/relationship between different immune cells. There are several factors that influence the diversity and distribution of lymphocyte subsets, such as race, gender, circadian rhythms, fertility status, lifestyle, and genetics, but first and foremost it is important to be aware of the development and maturation status of the immune system [6].

From mid-gestation and during the first months of life absolute T lymphocyte numbers increase, mainly because of the expansion of recent thymic emigrants and naïve cells, and after approximately 1.5 years gradually decrease to adult values [5, 7]. In the elderly T lymphocytes generally show progressive loss of functional capacity and cellular diversity, which reduces the immune response to antigens, cytotoxic activity of the cells, alters the cytokine secretion pattern, and accumulates memory lymphocyte subsets [8]. There is also an increase in the ratio of helper/cytotoxic T lymphocytes (Th/Tc) and in the ratio of memory/naïve T lymphocytes [9]. Double negative T lymphocytes which are mature postthymic T lymphocytes that lack the expression of CD4 and CD8 surface markers are absent at birth and expand with the advancing age. They have not been directly implicated in causing tissue damage; however, they are characteristically elevated in the subset of paediatric and adult patients with systemic autoimmune diseases such as autoimmune lymphoproliferative syndrome and systemic lupus erythematosus [10–12]. The mature, highly cytotoxic peripheral natural killer (NK) lymphocyte population increases with age, but its cytotoxic function declines, as does its ability to produce cytokines and chemokines, which might lead to a greater susceptibility of the aged to infection [8, 9, 13].

Based on their general function, Th lymphocytes can be roughly divided into effector Th lymphocytes (Teff) and regulatory Th lymphocytes (Treg). For a long time, the Teff response was divided solely into Th1 and Th2 lymphocytes [14]. The Th1/Th2 paradigm has been revised after the identification of another Th subset, namely, Th17 lymphocytes [15, 16]. The main function of Th17 lymphocytes seems to be the clearance of extracellular pathogens during infections by recruiting and activating neutrophil granulocytes [17]. They also secrete proinflammatory cytokine interleukin-17 (IL-17) and, to a lesser extent, tumor necrosis factor alpha (TNF-*α*) and IL-6 and have since been implicated in many autoimmune and infectious diseases such as hepatitis B and C virus infections, human immunodeficiency virus infection, cardiovascular diseases, inflammatory bowel disease, vitiligo, psoriasis, SLE, spondyloarthropathies, rheumatoid arthritis, types 1 and 2 diabetes, multiple sclerosis, atherosclerosis, Hashimoto's thyroiditis, and glioma [18, 19]. Recently, several studies have established the existence of human Th lymphocytes which are able to produce both interferon gamma (IFN-*γ*) and IL-17 and are thus called Th1Th17 lymphocytes [15, 17]. Accurate information on the exact dynamics of all the different Teff subsets with age is still lacking.

Treg lymphocytes suppress the proliferation, activation, and cytokine secretion of other immune cells, playing a central role in the maintenance of the peripheral self-tolerance, immune homeostasis, and tumour immunity [20, 21]. There are various conclusions regarding the changes in the proportion of circulating Treg lymphocytes with age and numerous diseases, probably partly due to differences in definitions of Tregs, although there is consensus that the absolute numbers remain small [7, 9, 22]. Immune tolerance maintains a fine balance between Teffs and Tregs. An inadequate number and function of Tregs or resistance of the Teff lymphocyte populations to suppression by Tregs can lead to the development of systemic autoimmune diseases such as systemic lupus erythematosus and rheumatoid arthritis [23, 24].

Recently a novel subset of CD4+FoxP3+Treg that does not express CD25 surface molecules (CD4+CD25−FoxP3+) has been identified [25]. Increased proportions of CD4+CD25−FoxP3+Treg are observed in particular in patients with systemic lupus erythematosus (SLE), especially in patients with lupus nephritis, a major cause of mortality and morbidity in SLE [26]. Furthermore, an age-related increase in the CD4+CD25−FoxP3+ cell population has been connected to a decline in T cell-mediated immune responses in mice; however, the origin, the evolution with age, the precise functional role, and the potential pathogenetic involvement of CD4+CD25−FoxP3+Treg in humans are still unknown [27].

Signal transducers and activators of transcription (STAT) are cytokine-induced signalling proteins, which are activated through phosphorylation and manage key immunological processes such as induction of tolerance, maintenance of homeostasis, and Th lymphocyte differentiation [28]. The primary role of STAT1 in vivo is to mediate IFN signalling and its antiviral and inflammatory effects. Aberrant baseline STAT1 phosphorylation has been associated with inflammatory diseases in humans and mice and may also be involved in ageing [29–31]. STAT5 proteins are essential mediators of common gamma chain cytokine (including IL-2) signalling in T lymphocytes, responsible for their survival, proliferation, differentiation, cell cycle progression, and homeostasis [32, 33]. The range of target genes which are under STAT5 regulation differs between cell subsets. The analysis of STAT5-target genes showed their imbalance may play a role in mechanisms leading to cancer [28, 34]. Persistent activation of STAT5 (pSTAT5) can also be involved in altering the epigenetic regulation of inflammatory response genes and ageing [29, 32, 35].

The purpose of this study was to analyse and compare different lymphocyte subsets in the peripheral blood of healthy children, adolescents, and adults. In addition to the routine immunophenotyping of lymphocyte subpopulations, we also evaluated the percentages of the subsets of effector and regulatory T lymphocytes as well as expression and basal phosphorylation of STAT1 and STAT5 proteins in Th lymphocytes. Lastly, we analysed specific Jak/STAT signalling response in the subsets of FoxP3+ Th lymphocytes in terms of their IL-2 induced STAT5 phosphorylation and STAT1 expression in otherwise healthy adult individuals.

Assessment of distinct lymphocyte subsets in healthy individuals can provide valuable data for subsequent comparative studies of various pathological conditions and could add to the knowledge of the immunological changes associated with ageing.

## 2. Materials and Methods

### 2.1. Study Subjects

Blood samples for analysing age-dependent changes in lymphocytes were obtained from sixty healthy subjects with no history of allergy, acute infections, autoimmune disorders, or medications that affect the immune system. They were divided into three groups: (1) twenty healthy children with a mean age of 4.2 years (range 10 months to 11.4 years), (2) twenty healthy adolescents with a mean age of 17.2 years (range 15.5 to 20.9 years), and (3) twenty healthy adults with a mean age of 51.2 years (range 40.8 to 63.3 years). Blood samples for the analysis of intracellular cytokine signalling were obtained from 6 healthy medical personnels. The study was approved by The National Medical Ethics Committee of the Republic of Slovenia; each participant or their guardian gave their informed consent.

### 2.2. Antibodies and Sample Staining for Analysis of Lymphocyte Subpopulations

Whole venous blood was collected into vacutainer tubes containing EDTA. The samples were processed for flow cytometry using three different methods. For surface staining, the standard whole blood staining methodology as prescribed by the manufacturer (BDBiosciences) was used. Anti-human monoclonal antibodies were combined in different multicolour panels. Panels of antibodies were as follows: anti-CD3−FITC/anti-CD8−PE/anti-CD45−PerCP/anti-CD4−APC (lymphocytes T, Th, and Tc), anti-CD3−FITC/anti-CD16+56−PE/anti-CD45−PerCP/anti-CD19−APC (lymphocytes B and NK cells), anti-CD45−FITC/anti-CD3−PE/anti-HLA-DR−APC (activated lymphocytes T), and anti-CD161−FITC/anti-CCR6−PE/anti-CD4−PerCP/anti-CXCR3−APC/anti-CCR4−Pe-Cy 7 (Teff lymphocytes). STAT expression and phosphorylation were studied following the BD Phosflow protocol (BDBiosciences), using formaldehyde containing red cell lysing solution (Phosflow Lyse/Fix Buffer, BDBiosciences) and methanol based buffer for permeabilisation (Perm buffer III, BDBiosciences). The staining of surface and intracellular antigens was done at the same time, using premixed antibody combinations: anti-CD3−FITC/anti-STAT1−PE/anti-pSTAT5(pTyr694)−Alexa Fluor 647/anti-CD4−PE-Cy 7 (STAT1, pSTAT5 proteins) or anti-STAT5−FITC/anti-CD3−PerCP/anti-pSTAT1(pTyr701)−Alexa Fluor 647/anti-CD4−PE-Cy 7 (pSTAT1, STAT5 proteins). For detecting regulatory T lymphocytes samples of whole blood were first stained for surface antigens with a mix of anti-CD25−PE/anti-CD45RA−APC/anti-CD4−PE-Cy 7, then fixed and permeabilised using Human FoxP3 Buffer set (BDBiosciences), and lastly stained for intracellular protein FoxP3, using anti-FoxP3−Alexa Fluor 488.

All antibodies were obtained from BDBiosciences (Mountain View, Ca, USA) except for anti-CD3−PerCP, anti-STAT5-FITC, pSTAT5-Alexa Fluor 647 (antibodies-online GmbH, Aachen, Germany), anti-CXCR3−APC (BioLegend Inc., San Diego, Ca, USA), and anti-CD161−FITC (eBioscience Inc., San Diego, Ca, USA). Cells were analysed on the FACSCantoII Flow Cytometer (BDBiosciences) equipped with blue (488 nm solid-state) and red (633 nm helium-neon) lasers. Digital data were acquired with the FACSDiva software (BDBiosciences) and analysed using the FlowJo software (Tree Star Inc.).

### 2.3. Flow Cytometric Analysis of Lymphocyte Subpopulations

**Figure 1:**
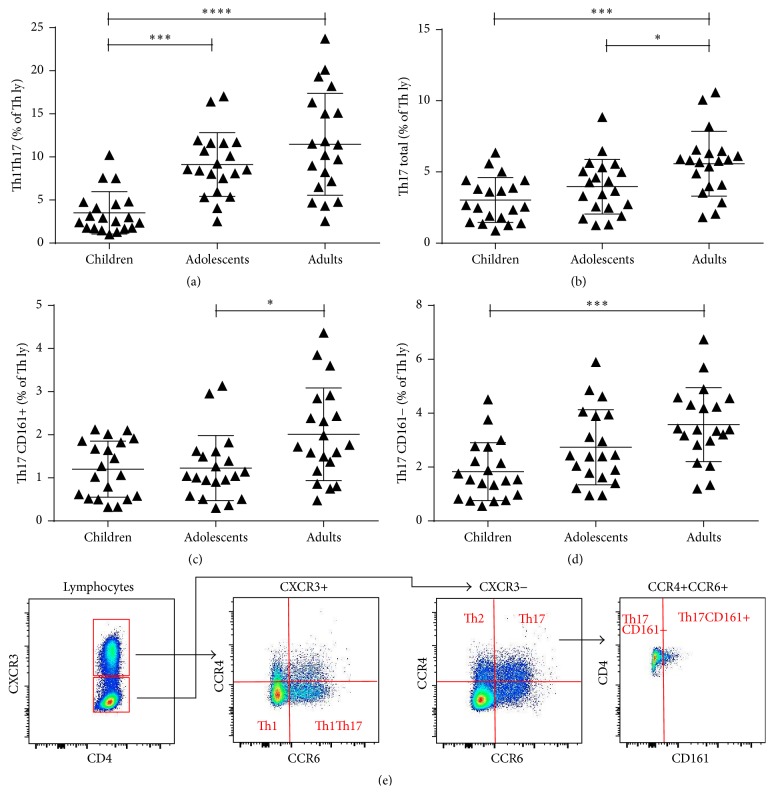
Th17 subset difference among populations. (a) The percentage of Th1Th17 lymphocytes in children was lower than those of adolescents or adults (b). Adult subjects had a higher percentage of total Th17 lymphocytes compared to both children and adolescents. (c) The increase in the percentage of Th17CD161+ lymphocytes in adults was significant compared to adolescents, and (d) the percentage of Th17CD161− lymphocytes in adults differed significantly from that in children. Each symbol in graphs (a) to (d) represents a single sample. The black lines in graphs (a) to (d) indicate the mean value with standard deviations. ^*∗*^ *P* < 0.05; ^*∗∗*^ *P* < 0.01; ^*∗∗∗*^ *P* < 0.001; ^*∗∗∗∗*^ *P* < 0.0001. (e) Gating strategies and staining patterns for identification of subpopulations Th1, Th2, Th1Th17, Th17CD161+, and Th17CD161−.

**Figure 2:**
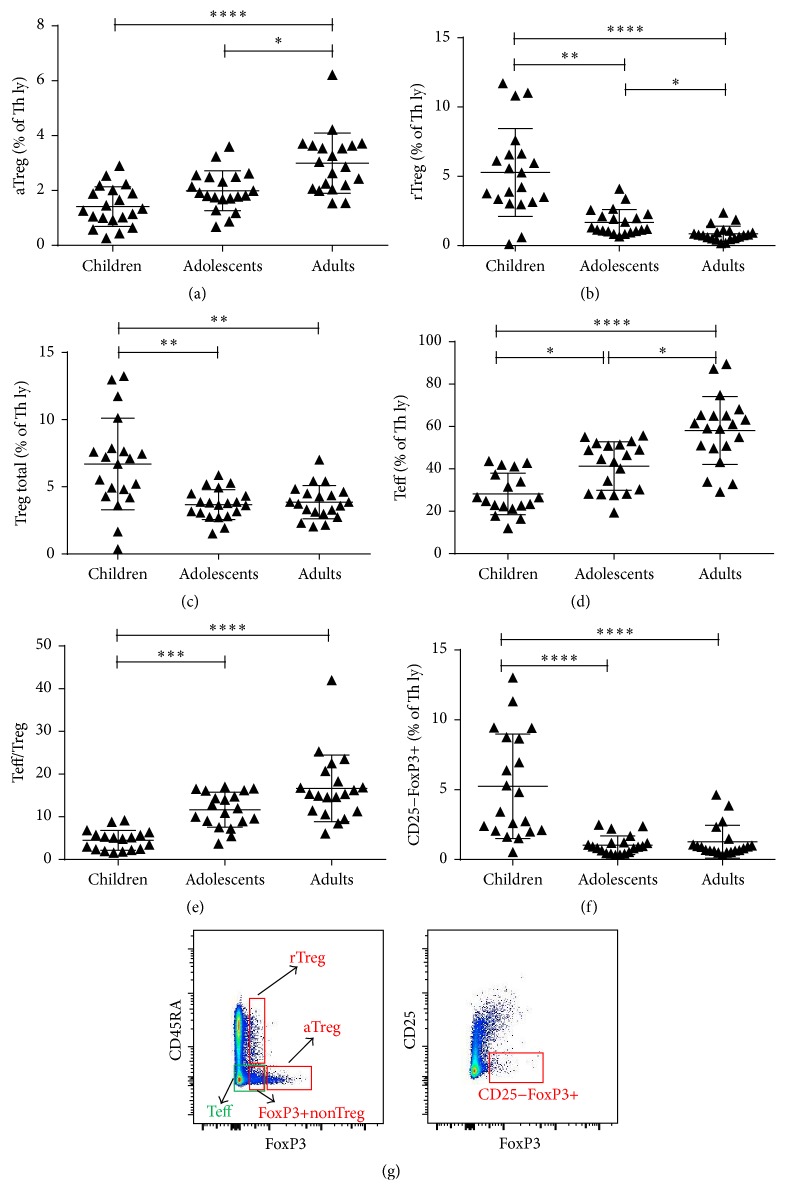
Treg lymphocyte subset difference among populations. (a) The percentage of aTreg lymphocytes was higher in adults compared to children as well as adolescents. (b) The percentage of rTreg lymphocytes decreased with age. There was a significant difference between all three subject groups. (c) The percentage of total Treg lymphocytes dropped with age as well, but there was no significant difference between the groups of adolescents and adults. (d) The percentage of Teff lymphocytes escalated significantly with age. (e) The ratio between Teff and Treg lymphocytes increased with age with a significant difference between children and adolescents as well as between children and adults. (f) The percentage of CD25−FoxP3+ Th lymphocytes dropped significantly from childhood to adolescence and stayed low in adults. Each symbol in graphs (a) to (d) represents a single sample. The black lines in graphs (a) to (d) indicate the mean value with standard deviations. ^*∗*^ *P* < 0.05; ^*∗∗*^ *P* < 0.01; ^*∗∗∗*^ *P* < 0.001; ^*∗∗∗∗*^ *P* < 0.0001. (g) Gating strategies and staining patterns for identification of subpopulations aTreg, rTreg, FoxP3+nonTreg, Teff, and CD25−FoxP3+.

To analyse the percentages of T, B, and NK cells, initial gating was on leucocytes and then lymphocytes. The percentages of Th, Tc, and activated T lymphocytes were analysed after subsequent gating on CD3+ cells. Absolute T cell counts were calculated using a dual-platform approach with panleukogating after measuring leukocyte (WBC) concentrations on Beckman Coulter AcT 8 Hematology Analyzer (BDBiosciences). For the analysis of all Th subsets, we gated lymphocytes on CD4+ cells. We then used gating on different characteristic combinations of surface or intracellular markers to identify and analyse each subset. Specific gating for Th17 subsets can be found in Figure 1(e) and for Treg subsets in Figure 2(g). For measurement of STAT proteins expression and basal phosphorylation levels, we gated lymphocytes on CD3+CD4+Th lymphocytes and then gated them on different STAT or pSTAT positive cells. The exact antibodies combination used to identify each of the analysed subsets can be found in Table 1.

Median fluorescent intensity (MFI) was used to measure expression levels and phosphorylation of STAT proteins. Samples were obtained and studied individually. For consistency we used the standard calibration beads (BD) to set the forward and side scatter and PMT voltage before each experiment. To account for interassay variability, the MFI of STAT1, STAT5, pSTAT1, and pSTAT5 in Th lymphocytes was normalized by subtraction of the MFI values of CD3−CD4− cells in each of the samples (ΔMFI).

### 2.4. Assessment of STAT5 Phosphorylation Responses after Ex Vivo IL-2 Stimulation

100 *μ*L of whole blood was incubated with 0.1 *μ*g/mL recombinant human IL-2 (Peprotech) or left untreated. After incubation for 15 min at 37°C in a water bath, induction of phosphorylation, cells were prepared as described above for STAT5 signalling analysis and stained with anti CD45RA−FITC/anti-FoxP3−PE or just anti FoxP3−Alexa Flour 488 antibodies together with anti CD45 PerCP/anti-pSTAT5−Alexa Flour 647/anti-CD4−Pe-Cy 7 in both cases (all BD Biosciences). Samples were analysed on the LSR II (BD Biosciences) flow cytometer, equipped with a 488 nm solid-state laser and a 633 nm helium-neon laser. The analysis of data was performed using the FACSDiva software (BDBiosciences) and FlowJo (Tree Star). To quantify changes in the levels of phosphorylation after IL-2 stimulation, the MFI of the pSTAT5 specific signal was measured and fold changes in phosphorylation were calculated as the ratio of MFI in stimulated versus unstimulated cells.

### 2.5. Analysis of Cytoplasmic and Nuclear pSTAT5

Whole blood samples were prepared and stained for CD3, CD25, and pSTAT5 as described above using anti-CD3−FITC, anti-CD25−PE, and anti-pSTAT5(pTyr694)−Alexa Flour 647 antibodies and counterstained with 20 ng/mL 7-AAD (all BD Biosciences). Image files of 50,000–100,000 events were collected for each sample using the ImageStreamX imaging flow cytometer (Amnis, Seattle, WA, USA) and analysed using the IDEAS software (Amnis) as described before [36]. In-focus single cells were identified by gating on 7-AAD-positive events with high nuclear aspect ratios (minor to major axis ratio, a measure of circularity) and high nuclear contrast (as measured by the Gradient Max feature). Among lymphocytes, CD25+ T lymphocytes were gated. Nuclear localization of pSTAT5 within these cells was measured using the Similarity score, which quantifies the correlation between pixel values of the nuclear and phosphotyrosine-STAT5 images on a per-cell basis [37]. If the transcription factor is nuclear, the two images will be similar and have large positive values. If it is cytoplasmic, the two images will be antisimilar and have large negative values.

### 2.6. Flow Cytometric Analysis of STAT1 Expression in Different Th Lymphocyte Subpopulations

Whole venous blood was collected into vacutainer tubes containing EDTA. STAT1 expression was studied following the BD Phosflow protocol as described above. STAT1 expression in FoxP3+ and FoxP3− Th lymphocytes was analysed using a panel of anti-CD45RA−FITC/anti-FoxP3−PE or just anti FoxP3−Alexa Flour 488 antibodies together with anti-CD45 PerCP/anti-STAT1−Alexa Flour 647/anti-CD4−Pe-Cy 7 antibodies in both cases. All antibodies were obtained from BD Biosciences. The samples were analysed on the LSR II (BD Biosciences) flow cytometer, equipped with a 488 nm solid-state laser and a 633 nm helium-neon laser. The analysis of data was performed using the FACSDiva software (BDBiosciences) and FlowJo (Tree Star).

### 2.7. ScatterSlice Analysis

Cells in EDTA anticoagulated whole blood samples were stained with anti-CD25PE antibodies and then prepared as described above for analysis of T-cell STAT signalling and costained with anti-CD3−FITC/anti-pSTAT5(pTyr694)−Alexa Flour 647/anti-CD4−PE-Cy 7 antibodies (all BD Biosciences). Initial analysis of flow cytometry data was performed using the FlowJo software (TreeStar). Data corresponding to CD4+ Th lymphocytes were gated/identified and exported as text files for analysis by processing R program ScatterSlice (http://www.scatterslice.org/). The details of the algorithm have been described before [38]. Text files are imported into R and then divided into user-specified bins. Within each bin, the geometric mean fluorescence intensity of the response channel (pSTAT5 in our studies) is calculated for display and further analysis.

### 2.8. Statistical Analysis

Differences across age groups were tested by means of the Kruskal-Wallis test for the three independent groups, with Dunn's multiple comparisons test if a significant effect was found. Correlations between the percentages of different Th lymphocyte subpopulations and STAT1 expression were examined using Pearson's correlation coefficient and Spearman's rank statistical tests. Differences between STAT5 phosphorylation and STAT1 expression for different FoxP3+ Th lymphocyte subpopulations were calculated using the Wilcoxon matched-pairs signed-rank test. A value of *P* < 0.05 was considered significant in all statistical tests. The statistical data analysis was performed using the GraphPad Prism software (GraphPad Software Inc., La Jolla, CA, USA).

## 3. Results

### 3.1. Maturation of T, B, Th, Tc, Double Negative T Lymphocytes, and NK Cells

Table 2 gathers differences in the percentages of all the studied lymphocyte subpopulations between healthy children, adolescents and adults, and cell counts of general lymphocyte subsets. The percentage of B lymphocytes decreased with age and was significantly higher in children (20.7%) than in adolescents (10.9%) or adults (9.4%; *P* < 0.0001 for both). In contrast to the percentage of B cells, we observed a gradual increase in the percentage of T lymphocytes with age. The difference was statistically significant between children and adolescents (60.0% versus 68.2%; *P* < 0.05) and between children and adults (60.0% versus 69.3%; *P* < 0.01). NK cells as a fraction of all lymphocytes were significantly higher in the adult age group (16.4%) compared to children (11.1%; *P* < 0.05). When comparing cell counts of major lymphocyte subpopulations, the differences in NK cells and T lymphocytes lost their statistical significance and only the concentration of B lymphocytes was significantly higher in children (0.60 × 10^9^/mL) compared to adolescents or adults (0.17 × 10^9^/mL for both *P* < 0.01 and *P* < 0.001, resp.).

There was no significant correlation between the proportion of Tc lymphocytes, HLA-DR+ T lymphocytes or the Th/Tc ratio, and subject age. The percentage of Th lymphocytes was significantly lower in children (54.4%) than in adults (62.9%; *P* < 0.01). The most significant difference in the percentage of T lymphocyte subsets was an inverse correlation between double negative T lymphocytes (CD3+CD4−CD8−) and subject age, comparing children (10.1%) with adults (4.4%; *P* < 0.0001) and comparing adolescents (7.8%) with adults (4.4%; *P* < 0.01). Concentrations of all T lymphocyte subpopulations can be found in Supplementary Material (see Additional file 1: Table S1 in Supplementary Material available online at http://dx.doi.org/10.1155/2015/352934).

### 3.2. Expansion of Subpopulations of Teff Lymphocytes with Age

We found a significantly decreased percentage of Th1 lymphocytes in children (10.6%) compared to adolescents (15.8%; *P* < 0.01) or adults (19.2%; *P* < 0.0001). Moreover, the percentage of Th2 lymphocytes was significantly decreased in children (3.4%) compared to adults (5.6%) and in adolescents (3.5%) compared to adults (both *P* < 0.05). No significant change in the Th1/Th2 ratio with age was observed. The percentage of Th1Th17 lymphocytes increased significantly, from 3.51% in children to 9.21% in adolescents and 11.47% in adults (Figure 1(a)). In addition, the percentage of total Th17 lymphocytes increased with age and was significantly higher in adults (5.6%) than in children (3.0%) and adolescents (4.0%) (Figure 1(b)). There was also a significant increase with age in the percentages of both Th17CD161+ and Th17CD161− subpopulations, with a greater increase in the latter subgroup (Figures 1(c) and 1(d)).

### 3.3. Decrease of Treg Lymphocytes with Age

No significant distinction in the percentage of FoxP3+non-Tregs (CD4+CD45RA−FoxP3lo) between groups was found. The percentage of activated Tregs (aTregs) which were defined as CD4+CD45RA−FoxP3hi increased significantly with age and was significantly higher in adults (3.0%) compared to children (1.4%) or adolescents (2.0%) (Figure 2(a)). Even more substantial was the decrease in the percentage of resting Tregs (rTregs) (CD4+CD45RA+FoxP3lo) with age, where we found a significant gradual decrease in percentages from children (5.3%) to adolescents (1.7%) and adults (0.9%) (Figure 2(b)). Altogether, the percentage of total Tregs (aTregs+rTregs) decreased significantly with age, from 6.7% in children to 3.7% in adolescents and 3.9% in adults (Figure 2(c)). CD4+CD45RA−FoxP3− lymphocytes were, together with FoxP3+non-Tregs, in this analysis functionally grouped as Teff lymphocytes. In contrast to Tregs we found a significant increase in the percentage of CD4+CD45RA−FoxP3− lymphocytes with age (Figure 2(d)), from 29.6% in children to 42.6% in adolescents and 58.13% in adults. This altogether led to an increase in the Teff/Treg ratio (Figure 2(e)), from 4.3% in children to 11.7% in adolescents and 16.7% in adults. We also found a significant decrease in the percentage of CD4+CD25−FoxP3+ lymphocytes, which dropped from 5.2% in children to 1.0% in adolescents and 1.3% in adults (Figure 2(f)).

### 3.4. STAT1 and STAT5 Protein Expression and Basal Phosphorylation in Unfractionated Th Lymphocytes and STAT1 Correlation with Percentages of Th Lymphocyte Subsets in Children and Adults

**Figure 3:**
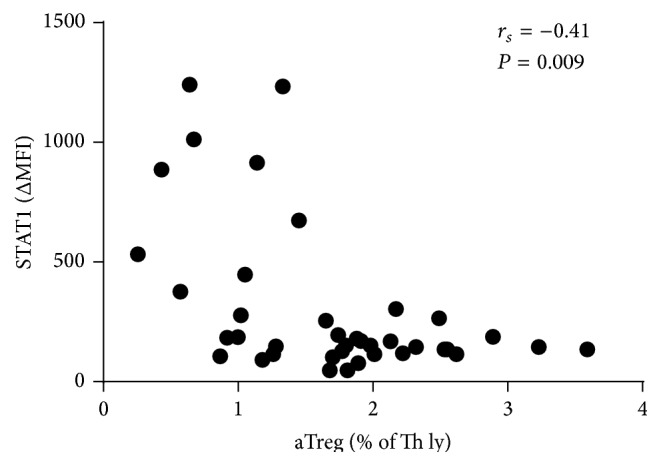
Correlation between STAT1 expression in Th lymphocytes and percentage of aTreg in group containing children and adults. A significant negative correlation was found between STAT1 protein expression in Th lymphocytes and the percentage of aTreg lymphocytes (*n* = 40). Each symbol represents a single sample. A value of <0.05 was considered significant.

In general STAT1 and STAT5 protein expression and basal phosphorylation in Th lymphocytes showed no significant differences between age groups. The only significant difference was found between the expression of STAT1 in children (ΔMFI = 422.6) and adolescents (ΔMFI = 186.7); therefore, we examined the relationship between STAT1 expression and the percentages of different subsets of Th cells. We found a significant negative correlation between Th lymphocyte STAT1 protein expression and the percentage of aTregs (Spearman correlation coefficient *r*_*s*_ = −0.41, *P* = 0.009) (Figure 3).

### 3.5. Differences in IL-2 STAT5 Phosphorylation Responses and STAT1 Expression between Subsets of Th Lymphocytes

Since differences in aTreg and FoxP3+ Th subsets lacking CD25 (IL-2Ralpha) expression were found, we additionally assessed the differences in IL-2 STAT5 phosphorylation responses and STAT1 protein expression in different Th lymphocyte subsets in the subgroup of healthy medical personnel.

**Figure 4:**
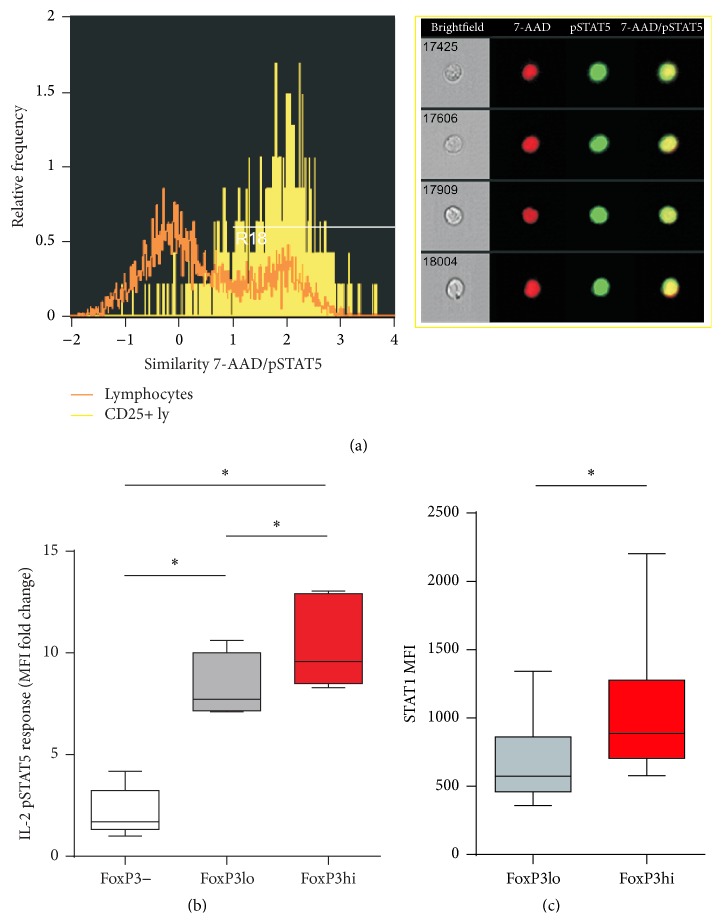
Th subset differences in STAT5 phosphorylation responses and STAT1 protein expression. (a) Colocalization of pSTAT5 within the 7-AAD stained nucleus after IL-2 stimulation in CD25+ T lymphocytes quantified by imaging flow cytometry as described in Section 2. Left: representative histograms of 7-AAD/pSTAT5. Similarity scores correlating 7-AAD nuclear stain with the pSTAT5 signal in CD25+ T lymphocytes (yellow) as compared to all lymphocytes (orange) gated on FoxP3 versus pSTAT5 dot plot are shown on the left. The higher the Similarity score is, the more the translocation is visualized in the example images of cells shown on the right: pSTAT5 (green) is specifically localized to the nucleus stained with 7-AAD (red) in CD25+ T lymphocytes. (b) pSTAT5 response to IL-2 stimulation in whole blood CD4+ Th lymphocytes and their FoxP3+ and FoxP3− subsets from healthy donors was assayed by measuring median fluorescence intensities (MFI) and represented as a ratio of induction to baseline levels (MFI fold change = MFI cytokine-stimulated/MFI unstimulated cells). Box and whisker plots of pSTAT5 response after IL-2 stimulation in FoxP3hi expressing aTregs as compared to FoxP3lo and FoxP3− Th subsets from healthy donors (*n* = 7). (c) Box and whisker plots of STAT1 protein expression in FoxP3hi expressing aTregs as compared to FoxP3lo expressing Th subsets from healthy donors (*n* = 6). *P* values were calculated using the Wilcoxon matched-pairs signed-rank test. ^*∗*^ *P* < 0.05; ^*∗∗*^ *P* < 0.01.

Phosphorylation of STAT5 protein was induced in the CD25+ subset of whole blood T lymphocytes from healthy donors after IL-2 stimulation. Scatter-slice analysis showed higher STAT5 phosphorylation levels after IL-2 stimulation in the high IL-2Ralpha expressing population of Th lymphocytes, confirming the contingency of STAT5 response and the level of IL-2Ralpha expression (Additional file 2: Figure S1). Imaging flow cytometry also confirmed a successful nuclear location of phosphorylated STAT5 after IL-2 stimulation in CD25+T lymphocytes (Figure 4(a)). We found that IL-2 STAT5 phosphorylation responses were significantly higher in FoxP3hi aTregs than other—FoxP3lo and FoxP3− subsets of CD4+ T lymphocytes. The MFI fold change comparing the unstimulated and the stimulated was 9.58 for FoxP3hi (aTregs), 7.72 for FoxP3lo, and 1.69 for FoxP3− Th lymphocytes (Figure 4(b)).

Significant differences in STAT1 expression were found also between the FoxP3hi aTreg (1045.3 MFI) and FoxP3lo (672.8 MFI) subsets of CD4+ T-cells (Figure 4(c)).

## 4. Discussion

In the present study, an extended lymphocyte subset analysis was performed including effector and regulatory T lymphocytes as well as expression and basal phosphorylation of STAT1 and STAT5 proteins in Th cells. To our knowledge, this study was the first in which analysis of effector and regulatory subsets of T lymphocytes and expression/phosphorylation of intracellular signalling proteins was evaluated in healthy subjects of a broad age range, from 10 months to 63 years. We also performed a series of analyses for a better understanding of STAT1 and STAT5 expression and activation patterns in subgroups of Th in healthy adults.

Our results demonstrate children having a significantly higher percentage of B lymphocytes than adolescents and adults but lower percentages of T lymphocytes and NK cells. This is in agreement with some of the previous reports highlighting a functional decline and decrease in diversity in ageing B cells along with their shift from naïve to memory cells [2, 13, 39, 40]. The observed increase in the percentages of NK cells and T lymphocytes with age is a direct consequence of this rapid change in the number of B cells in childhood, especially in the first years of life, and does not reflect any major changes in the number of NK cells and T lymphocytes themselves (as the percentage of B lymphocytes decreases, the percentage of other cells among total lymphocytes naturally increases); therefore, we found no significant difference in the concentrations of NK cells and T lymphocytes amongst the three groups.

There was a significantly higher percentage of Th lymphocytes in adult subjects, while there was no significant difference in the percentages of Tc lymphocytes, activated T lymphocytes (HLA-DR+), or the Th/Tc (CD4+/CD8+) T lymphocyte ratio. Information regarding these general lymphocyte subpopulations in the literature is conflicting and seems to be at least partially dependent on ethnicity, socioeconomic status, average diet, and so forth, of the studied cohort [9, 22, 40–43]. We found a significant decrease in the percentage of CD4−CD8− double negative lymphocytes with age. These cells have been studied mostly in pathological conditions and were characteristically increased in patients with lymphoproliferative syndrome and systemic lupus erythematosus [10–12].

Subpopulations of Teff lymphocytes are generally studied by secretion of characteristic cytokines such as IFN-*γ* for Th1, IL-4 for Th2, and IL-17 for Th17 cells. Crome et al. described a method for the isolation and identification of in vivo differentiated Th17 using unique patterns of surface CD receptors [44]. The use of characteristic combinations of these receptors allows for surface staining without the need for extensive stimulation, fixation, and permeabilisation of the cells, which enables simple and precise flow cytometry analysis of different cell subpopulations and was therefore used for the identification and analysis of Th1, Th2, Th1Th17, and Th17 lymphocytes also in our study. Staining patterns and gating practices we used are shown in representative flow cytometric images in Figure 1(e). The analysis of these chosen Teff subsets showed a significant increase in both Th1 and Th2 lymphocytes with age but no significant difference in the Th1/Th2 ratio between the three age groups. One of the most remarkable findings of the present study was a significant increase in the percentage of Th1Th17 cells in adults. These cells, identified by expressing both chemokine receptor CXCR3 (CD183) and CCR6 (CD196, the receptor for CCL20 and *β*-defensins), were reported to secrete IL-17 as well as IFN-*γ*, combining characteristic responses of Th1 and Th17, suggesting a possible common origin between human Th17 and Th1 lymphocytes [15, 17]. Th1Th17 cells were reported to show a considerable ability to help B cells and reduced susceptibility to suppression by autologous CD4+FoxP3+ regulatory T lymphocytes, thus supporting the switch from homeostasis towards inflammation [15, 45]. The increase in Th1Th17 cells could therefore contribute to the development of inflammatory and autoimmune diseases with age. Moreover, our study also showed a significant increase in the percentages of total Th17, Th17CD161+, and Th17CD161− lymphocytes with age. CD161 was shown to be an important marker of IL-17 expressing cells, but even though Th17CD161– cells express significantly less IL-17 than Th17CD161+ cells, they still express enough of it to be considered functional Th17 cells and as such proinflammatory in vivo [17, 44]. A general increase in the proportions of Teff lymphocytes has been implicated in many diseases, but the results of our study imply ageing alone could be the reason for the shift in homeostasis and the expansion of proinflammatory cells [18, 19].

Discrepancies in the reported stagnancy or increase of Treg with age can be contributed to a different methodology of identifying the Treg subset, focusing only on high expression of CD25 and transcriptional factor FoxP3 and not considering the functional capacity of the selected cells [9]. Even though CD25 is indeed highly expressed in regulatory T lymphocytes it also appears to be upregulated in the majority of activated human CD4+ T cells; similarly, not all CD4+FoxP3+ T cells can be considered anergic immunosuppressive in humans [46]. Different identification and gating strategies can therefore inadvertently include various cell subsets which are phenotypically not true Tregs and do not contribute to the maintenance of peripheral tolerance [21, 47, 48].

In our study, Treg lymphocytes were identified using CD4, CD25, CD45RA, and FoxP3 markers (Figure 2(g)) and according to Miyara et al. split in three subsets: activated Tregs, resting Tregs, and cytokine-secreting nonrepressive FoxP3+non-Tregs [21]. The percentage of total functionally suppressive Treg lymphocytes (aTregs + rTregs) as well as the percentage of rTregs decreased significantly with age, while the percentage of aTregs increased significantly (Figures 2(a), 2(b), and 2(c)). Our results of this inverse correlation between aTergs and rTregs are in agreement with the paradigm of a general shift of immune cells from naïve towards activated and memory phenotype and a general decrease of total Tregs with age [13, 20, 22]. The maintenance of Treg lymphocytes in the periphery is a dynamic process influenced not only by normally functioning thymus but also by genetic factors, proliferation rate of Treg lymphocytes, and movement of activated Treg lymphocytes to inflammatory sites, where differentiated Tregs eventually die. The latter process could explain the decline in the amount of circulating Tregs, which has been observed in patients with atopic allergies, food allergies, chronic graft-versus-host disease, and, in particular, various systemic autoimmune diseases such as primary antiphospholipid syndrome, rheumatoid arthritis, and systemic lupus erythematosus [21, 23, 33, 46, 49, 50].

To be able to make a reliable comparison between the percentages of total Treg and total Teff, we used results from sample staining in the same tube to define both groups. Total Teffs were in this instance considered a group consisting of effector-like cytokine-secreting FoxP3+non-Tregs, combined with CD4+CD45RA−FoxP3− lymphocytes, as described before [48]. We found no significant difference in the percentage of FoxP3+non-Tregs with age. Nevertheless, the total percentage of total Teff increased significantly with age (Figure 2(d)). The impact of a decrease in the proportion of phenotypically suppressive Tregs (i.e., totalTregs) is even greater when coupled with an increase in cytokine producing Teffs and can have a considerable effect on overall immune balance and eventually contribute to a state of diminished immune status in the elderly. In our study, these changes are additionally demonstrated by the dramatic increase in the Teff/Treg ratio (Figure 2(e)).

Next, age-dependent dynamics of a novel Treg subset—CD4+CD25−FoxP3+ lymphocytes—were evaluated, which had previously not been studied in humans. We found a significant decrease in the percentage of these cells with age, specifically from childhood to adolescence (Figure 2(f)). This is of special interest as there is a link between increased CD4+CD25−FoxP3+ lymphocytes and lupus nephritis [26]. A higher percentage of CD4+CD25−FoxP3+ lymphocytes in the paediatric population overall could explain more frequent renal involvement at the time of diagnosis experienced in paediatric patients with SLE compared to adult patients [51].

In addition to the analysis of age-associated differences in the heterogeneity of human Th lymphocytes we were also interested in their STAT-dependent signal transduction. Levels of expression and basal phosphorylation of proteins STAT1 and STAT5 are indicators of IFN and common gamma chain cytokine (IL-2 and/or IL-7 and others) dependent activation and even priming (i.e., enhancing responsiveness to other inflammatory stimuli) of the cells [52, 53]. There is emerging evidence of general IFN signature in autoimmune disorders as well as a direct pathogenic role of overexpression and phosphorylation of STAT1 and STAT5 in mice and humans [30, 31, 35, 54]. Therefore, we assessed if this aberrant cytokine signal transduction, seen in pathological conditions, can also be observed in unfractionated Th lymphocytes with ageing. The only significant difference between all three groups in expression and basal phosphorylation of STAT1 and STAT5 in these cells was the expression of STAT1 in children, which was higher than in adolescents. This was associated with their lower percentage of aTregs (Figure 3), which showed the highest STAT1 expression among FoxP3+CD4+ T-cells in our subsequent analysis (Figure 4(c)). Of note, aTregs had been shown before to be more prone to in vitro IFN-alpha mediated suppression than Teff cells [55]. Serum IFN-alpha activity is higher in younger individuals in the SLE family cohorts, and this tendency is accentuated in affected individuals [56]. This age-related pattern of IFN-alpha activity may contribute to perturbed aTreg homeostasis in SLE, which has been described before, and the increased incidence of this disease in early adulthood [46].

Even though our study subjects covered a substantial age range, from 10 months to 63 years, it would be interesting to examine more precisely changes in very young healthy children and the elderly as well. The immune system of children is known to undergo dramatic changes in the first years and even months of their lives and should therefore ideally be grouped and studied in several age groups with a small age range rather than in one general group.

Cell subset specific variations in induced immune signalling responses had been shown in Th lymphocytes from healthy donors before [57]. To quantify the impact of IL-2R subunit alpha (CD25) expression, which forms a high-affinity receptor for IL-2 on IL-2 induced pSTAT5, we used scatter-slice analysis [38, 58]. This analysis revealed that such STAT5 phosphorylation response in Th lymphocytes from healthy donors was indeed dependent on the level of their CD25 expression as it was increased in CD25 expressing Th lymphocytes. As cytokine dependent activation/phosphorylation of STAT5 results in the translocation of STAT5 homodimers to the nucleus, we used imaging flow cytometry to examine and confirm pSTAT5 nuclear localization, hence the complete signal transduction in whole blood CD25+ T lymphocytes after IL-2 stimulation (Figure 4(a)) [59].

The FoxP3+ subset of Th lymphocytes is, in general, characterised by higher CD25 expression. Consistent with that, IL-2 induced pSTAT5 signalling response was significantly higher in FoxP3+ subsets than in the FoxP3− subset of Th lymphocytes from healthy donors (Figure 4(b)). However, significant IL-2 induced STAT5 phosphorylation differences were found even among the subsets of FoxP3+ Th lymphocytes (Figure 4(b)). As we found significant differences in the percentages (within Th lymphocytes) of (CD25−) Treg lymphocyte subpopulations with age, there could be age-associated changes in IL-2 induced STAT5 signalling in different Th lymphocyte subsets, even though STAT5 protein expression and basal phosphorylation in unfractionated Th lymphocytes showed no significant differences between age groups in our study.

Finally, higher expression of STAT1 was found in FoxP3hi (considered aTregs) than in FoxP3low Th lymphocytes. As discussed before, we proved a shift in the Teff/Treg ratio with ageing and an expansion of aTregs with age. Higher STAT1 protein expression in the most suppressive subset of Tregs, FoxP3hi (aTreg), could explain the higher IFN sensitivity of aTregs and the associated changes in their homeostasis with age, found in our study [55].

## 5. Conclusion

In summary, in the present study we observed an increase in the percentage of Teff lymphocytes with age. The raise was most apparent in Th1, Th1Th17, and Th17CD161− lymphocytes although it was still significant in Th2 and Th17CD161+ lymphocytes. Our results also demonstrate a decrease in the percentage of total Tregs and an increase in the percentage of total Teff with age and a consequential immense increase in the Teff/Treg ratio in adults. Interestingly, the percentage of CD4+CD25−FoxP3+ cells drops dramatically from childhood to adolescence. We found significant higher STAT1 protein expression in Th lymphocytes in children compared to adolescents; children with high Th lymphocyte STAT1 protein expression had a low percentage of activated Treg subset. Our study suggests a link between immune system maturation, ageing, and age-related changes in circulating Teff and Treg, as well as significant differences in STAT5/STAT1 signalling among FoxP3+ Th lymphocytes, which is worth further study.

## Supplementary Material

Table S1. Concentrations of remaining studied cell subsets in all three age groups of healthy subjects analyzed by flow cytometry (∗P < 0.05; ∗∗P < 0.01; ∗∗∗P < 0.001; ∗∗∗∗P < 0.0001). Calculated concentrations of the T lymphocyte subsets complement information showed by the percentages. There is similar significant increase in the concentrations of all different Teff subsets whereas the concentrations of the rTreg subset which is group of cells that is the most supressive in vivo significantly drops with age.Together this data demonstrate a significant shift towards an effector T cell phenotype as children become adults.
Additional file 2: Figure S1. pSTAT5 after IL-2 stimulation in Th lymphocytes depends on the levels of IL-2Ralpha. Whole blood samples were stimulated with IL-2, stained with anti-CD25 antibodies and prepared for analysis of Th cell STAT5 signalling as described in materials and methods. Representative plots of gated lymphocytes (A) and CD4+ Th cells (B) from healthy donors are shown. The small boxes represent individual bins within the distribution of CD25 (IL-2Ralpha) and CD4. Within each bin, the geometric mean fluorescence intensity of the response channel (pSTAT5) was calculated for display using ScatterSlice software: pSTAT5 for varying levels of IL-2Ralpha and CD4 as defined by colour code shown on the right.

## Figures and Tables

**Table 1:** Combination of antibodies used for identification and analysis of different lymphocyte subpopulations.

Lymphocyte subpopulations	Combination of antibodies
NK	CD3+CD16/CD56+CD45+
B ly	CD3−CD19+CD45+
T ly	CD3+CD45+
Th	CD3+CD4+CD45+
Tc	CD3+CD8+CD45+
DNT	CD3+CD4−CD8−CD45+
HLA-DR	CD3+CD45+HLA-DR+

Th1	CD4+CXCR3+CCR4−CCR6−
Th2	CD4+CXCR3−CCR4+CCR6−
Th1Th17	CD4+CXCR3+CCR4−CCR6+
Th17CD161+	CD4+CXCR3−CCR4+CCR6+CD161+
Th17CD161−	CD4+CXCR3−CCR4+CCR6+CD161−

aTreg	CD4+CD45RA−FoxP3hi
rTreg	CD4+CD45RA+FoxP3lo
FoxP3+non-Treg	CD4+CD45RA−FoxP3lo
Teff	CD4+CD45RA−FoxP3lo, CD4+CD45RA−FoxP3−
CD25−FoxP3+	CD4+CD25−FoxP3+

STAT5	CD3+CD4+STAT5+
pSTAT5	CD3+CD4+STAT5(pTyr694)+
STAT1	CD3+CD4+STAT1+
pSTAT1	CD3+CD4+STAT1(pTyr701)+

**Table 2:** Studied cell subsets in all three age groups of healthy subjects analysed by flow cytometry (^*∗*^ *P* < 0.05; ^*∗∗*^ *P* < 0.01; ^*∗∗∗*^ *P* < 0.001; ^*∗∗∗∗*^ *P* < 0.0001).

	Children^A^		Adolescents^B^		Adults^C^	
	*N*	Mean ± SD	*N*	Mean ± SD	*N*	Mean ± SD
Mean age	20	4.17 ± 3.22	20	17.24 ± 1.93	20	51.17 ± 6.32
NK (% of ly)	20	11.13 ± 4.66	20	16.25 ± 8.12	20	16.35 ± 5.79^AC⁡*∗*^
B ly (% of ly)	20	20.73 ± 6.84	20	10.85 ± 3.77^AB*∗∗∗∗*^	20	9.44 ± 4.60^AC⁡*∗∗∗∗*^
T ly (% of ly)	20	59.97 ± 7.39	20	68.19 ± 8.61^AB*∗*^	20	69.28 ± 7.96^AC⁡*∗∗*^
Th (% of T ly)	20	54.40 ± 8.30	20	58.22 ± 7.06	20	62.86 ± 10.92^AC⁡*∗∗*^
Tc (% of T ly)	20	35.02 ± 6.47	20	33.41 ± 6.71	20	31.70 ± 10.38
Th/Tc	20	1.64 ± 0.54	20	1.84 ± 0.54	20	2.30 ± 1.11
DNT (% of T ly)	20	10.08 ± 4.57	20	7.80 ± 2.57	20	4.39 ± 3.11^AC⁡*∗∗∗∗*,BC*∗∗*^
HLA-DR (% of T ly)	19	13.90 ± 8.02	20	15.66 ± 8.96	20	18.41 ± 7.67
Th1 (% of Th ly)	20	10.57 ± 3.00	20	15.79 ± 4.28^AB*∗∗*^	20	19.20 ± 5.82^AC⁡*∗∗∗∗*^
Th2 (% of Th ly)	20	3.43 ± 1.86	20	3.50 ± 1.26	20	5.62 ± 2.99^AC⁡*∗*,BC*∗*^
Th1/Th2	20	3.82 ± 1.81	20	5.16 ± 2.58	20	4.06 ± 1.73
Th1Th17 (% of Th ly)	20	3.51 ± 2.46	20	9.12 ± 3.70^AB*∗∗∗*^	20	11.47 ± 5.93^AC⁡*∗∗∗∗*^
Th17CD161+ (% of Th ly)	20	1.20 ± 0.65	20	1.23 ± 0.75	20	2.01 ± 1.08^AC⁡*∗*^
Th17CD161− (% of Th ly)	20	1.83 ± 1.07	20	2.74 ± 1.40	20	3.58 ± 1.38^AC⁡*∗∗∗*^
Th17 total (% of Th ly)	20	3.03 ± 1.58	20	3.96 ± 1.92	20	5.59 ± 2.27^AC⁡*∗∗∗*,BC*∗*^
aTreg (% of Th ly)	20	1.41 ± 0.72	20	1.99 ± 0.72	20	3.00 ± 1.10^AC⁡*∗∗∗∗*,BC*∗*^
rTreg (% of Th ly)	20	5.28 ± 3.17	20	1.68 ± 0.93^AB*∗∗*^	20	0.85 ± 0.55^AC⁡*∗∗∗∗*,BC*∗*^
FoxP3+non-Treg (% of Th ly)	20	2.95 ± 1.12	20	3.04 ± 1.12	20	3.23 ± 0.93
Treg total (% of Th ly)	20	6.69 ± 3.41	20	3.67 ± 1.11^AB*∗∗*^	20	3.85 ± 1.24^AC⁡*∗∗*^
Teff (% of Th ly)	18	26.69 ± 9.80	19	41.30 ± 11.47^AB*∗*^	20	58.13 ± 16.03^AC⁡*∗∗∗∗*,BC*∗*^
Teff/Treg	18	4.25 ± 2.34	19	11.66 ± 4.09^AB*∗∗∗*^	20	16.66 ± 7.80^AC⁡*∗∗∗∗*^
CD25−FoxP3+ (% of Th ly)	20	5.16 ± 3.74	20	1.03 ± 0.67^AB*∗∗∗∗*^	20	1.27 ± 1.18^AC⁡*∗∗∗∗*^

STAT5 (ΔMFI)	12	21.8 ± 29.7	12	16.10 ± 10.00	17	27.6 ± 36.9
pSTAT5 (ΔMFI)	20	240.4 ± 214.8	20	174.9 ± 107.5	20	228.4 ± 97.4
STAT1 (ΔMFI)	20	422.6 ± 372.5	20	186.7 ± 202.4^AB*∗∗*^	20	200.0 ± 62.3
pSTAT1 (ΔMFI)	7	13.4 ± 17.3	7	5.9 ± 5.3	14	32.4 ± 47.8

WB (×10^9^ mL^−1^)	20	7.56 ± 3.14	20	6.65 ± 1.50	20	6.90 ± 1.34
ly (×10^9^ mL^−1^)	20	2.79 ± 1.94	20	1.57 ± 0.48	20	1.80 ± 0.53
NK (×10^9^ mL^−1^)	20	0.33 ± 0.32	20	0.26 ± 0.18	20	0.30 ± 0.16
B ly (×10^9^ mL^−1^)	20	0.60 ± 0.47	20	0.17 ± 0.07^AB*∗∗*^	20	0.17 ± 0.10^AC⁡*∗∗∗*^
T ly (×10^9^ mL^−1^)	20	1.63 ± 1.11	20	1.07 ± 0.34	20	1.25 ± 0.38

A, B and C in the table are referring to our three different age groups (as it is indicated in the first row of the table A = children, B = adolescents, C = adults). Abbreviations AB, AC an BC are therefore used to highlight statistically significant differences between compared groups (AB = comparing children to adolescents, AC = comparing children to adults, BC = comparing adolescents to adults).
