# Supplementary material for: Age-Related Differences in Percentages of Regulatory and Effector T Lymphocytes and Their Subsets in Healthy Individuals and Characteristic STAT1/STAT5 Signalling Response in Helper T Lymphocytes
Source: J Immunol Res. 2015 Oct 7;2015:352934. doi: 10.1155/2015/352934 (PMC4615205; doi:10.1155/2015/352934)
Supplement: Supplementary file 1 — Table S1. Concentrations of remaining studied cell subsets in all three age groups of healthy subjects analyzed by flow cytometry (∗P < 0.05; ∗∗P < 0.01; ∗∗∗P < 0.001; ∗∗∗∗P < 0.0001). Calculated concentrations of the T lymphocyte subsets complement information showed by the percentages. There is similar significant increase in the concentrations of all different Teff subsets whereas the concentrations of the rTreg subset which is group of cells that is the most supressive in vivo significantly drops with age.Together this data demonstrate a significant shift towards an effector T cell phenotype as children become adults. Additional file 2: Figure S1. pSTAT5 after IL-2 stimulation in Th lymphocytes depends on the levels of IL-2Ralpha. Whole blood samples were stimulated with IL-2, stained with anti-CD25 antibodies and prepared for analysis of Th cell STAT5 signalling as described in materials and methods. Representative plots of gated lymphocytes (A) and CD4+ Th cells (B) from healthy donors are shown. The small boxes represent individual bins within the distribution of CD25 (IL-2Ralpha) and CD4. Within each bin, the geometric mean fluorescence intensity of the response channel (pSTAT5) was calculated for display using ScatterSlice software: pSTAT5 for varying levels of IL-2Ralpha and CD4 as defined by colour code shown on the right. [file 352934.f1.pdf]

Additional file provided with this submission

Additional file 1: Table S1. Concentrations of remaining studied cell subsets in all three age groups of healthy subjects analysed by flow cytometry (*P < 0.05; **P < 0.01; ***P < 0.001;**P<0.0001).**

	children^{A}				adolescents^{B}				adults^{C}			
	N	mean	±	SD	N	mean	±	SD	N	mean	±	SD
Th (x10^{9} mL^{-1})	20	0.90	±	0.63	20	0.61	±	0.19	20	0.76	±	0.20
Tc (x10^{9} mL^{-1})	20	0.57	±	0.42	20	0.36	±	0.16	20	0.42	±	0.23
DNT (x10^{9} mL^{-1})	20	0.15	±	0.10	20	0.08	±	0.04	20	0.05	±	0.04^{AC***}
HLA-DR (x10^{9} mL^{-1})	19	0.25	±	0.32	20	0.17	±	0.12	20	0.25	±	0.14
Th1 (x10^{9} mL^{-1})	20	0.088	±	0.054	20	0.100	±	0.055	20	0.147	±	0.052^{AC**,BC*}
Th2 (x10^{9} mL^{-1})	20	0.029	±	0.034	20	0.022	±	0.012	20	0.044	±	0.028^{AC*,BC**}
Th1Th17 (x10^{9} mL^{-1})	20	0.024	±	0.013	20	0.055	±	0.024^{AB***}	20	0.086	±	0.049^{AC****}
Th17 CD161^{+} (x10^{9} mL^{-1})	20	0.009	±	0.007	20	0.007	±	0.005	20	0.015	±	0.007^{AC*,BC**}
Th17 CD161^{-} (x10^{9} mL^{-1})	20	0.016	±	0.019	20	0.016	±	0.009	20	0.028	±	0.014^{AC***,BC*}
Th17 total (x10^{9} mL^{-1})	20	0.025	±	0.025	20	0.024	±	0.012	20	0.042	±	0.020^{AC***,BC**}
aTreg (x10^{9} mL^{-1})	20	0.010	±	0.006	20	0.012	±	0.005	20	0.022	±	0.009^{AC****,BC**}
rTreg (x10^{9} mL^{-1})	20	0.044	±	0.037	20	0.010	±	0.005^{AB*}	20	0.007	±	0.005^{AC****}
FoxP3+ non Treg (x10^{9} mL^{-1})	20	0.024	±	0.018	20	0.019	±	0.009	20	0.024	±	0.009
Treg total (x10^{9} mL^{-1})	20	0.054	±	0.040	20	0.022	±	0.007	20	0.029	±	0.011
Teff (x10^{9} mL^{-1})	18	0.214	±	0.127	19	0.243	±	0.091	20	0.434	±	0.148^{AC****,BC***}
CD25-FoxP3+ (x10^{9} mL^{-1})	20	0.039	±	0.034	20	0.006	±	0.005^{AB****}	20	0.010	±	0.010^{AC***}

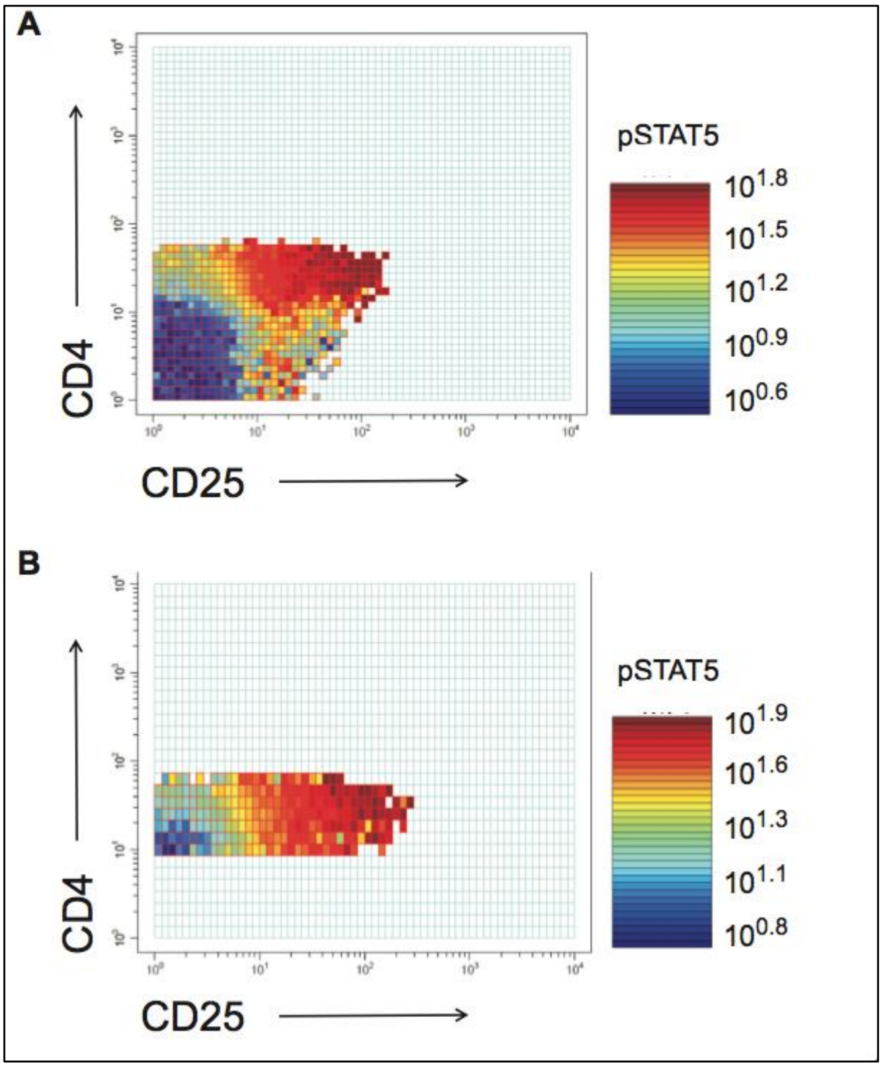

Additional file 2: Figure S1. pSTAT5 after IL-2 stimulation in Th lymphocytes depends on levels of IL-2Ralpha. Whole blood samples were stimulated with IL-2, stained with anti-CD25 antibodies and prepared for the analysis of Th cell STAT5 signalling as described in materials and methods. Representative plots of gated lymphocytes (A) and CD4+ Th cells (B) from healthy donors are shown. The small boxes represent individual bins within the distribution of CD25 (IL-2Ralpha) and CD4. Within each bin, the geometric mean fluorescence intensity of the response channel (pSTAT5) was calculated for display using the ScatterSlice software: pSTAT5 for varying levels of IL-2Ralpha and CD4 as defined by the colour code shown on the right.
